# Barriers and Facilitators to Psychological Safety During Medical Procedures Among Individuals Diagnosed with Chronic Illness in Childhood

**DOI:** 10.3390/healthcare13080914

**Published:** 2025-04-16

**Authors:** Hannah Roche, Liza Morton, Nicola Cogan

**Affiliations:** 1 Psychology Department, Glasgow Caledonian University, Cowcaddens Road, Glasgow G4 0BA, UK; hroche300@caledonian.ac.uk; 2 School of Psychological Sciences and Health, University of Strathclyde, Glasgow G1 1XQ, UK; nicola.cogan@strath.ac.uk

**Keywords:** psychological safety, chronic disease, patient-centred care, mental health, psychologically informed healthcare

## Abstract

**Background:** This study explores barriers and facilitators to psychological safety during medical procedures among individuals diagnosed with chronic illnesses in childhood. Psychological safety in healthcare, detected via neuroception and the autonomic nervous system’s responses to perceived safety or threat, is essential for the well-being and mental health of chronically ill patients, especially those with early diagnoses. **Methods:** Using Polyvagal Theory as a framework, semi-structured interviews were conducted with six participants (aged 20–64) who experienced chronic disease from a young age. The Neuroception of Psychological Safety Scale (NPSS) guided thematic exploration to understand participants’ experiences. Thematic analysis identified key themes that reflect contributors and detractors to psychological safety during medical care. **Results:** Four primary themes were developed: (1) knowledge empowerment through information and facilitated inquiry, (2) holistic acknowledgment of psychological and social impacts, (3) the role of parental involvement in healthcare interactions, and (4) the need for an individualised, patient-centred approach. Participants expressed a need for psychological support integrated with their medical treatment and the importance of autonomy and clear communication. **Conclusions:** Psychological safety is central to medical experiences for chronically ill individuals and requires a patient-centred, psychologically informed approach. Emphasising tailored support, family involvement, and comprehensive mental health consideration can foster more effective care and enhance patients’ long-term well-being.

## 1. Introduction

### 1.1. The Burden of Chronic Disease in Childhood

Chronic diseases, the leading cause of disability worldwide, are estimated to impact one third of the global population [1]. Defined by the World Health Organisation [2] as health conditions that are incurable and persistent in duration, they include a wide range of conditions such as cancer, diabetes, congenital and coronary heart disease, autoimmune disorders, asthma and mental health disorders [3]. Although chronic diseases are incurable, they can be managed through medication, lifestyle changes, education, and medical interventions such as surgery, depending on the condition [4]. While chronic diseases are a highly individualised experience, most are associated with the “patient burden” [5], which typically includes self-management of the condition, adherence to treatment regimens, attending medical appointments and associated administrative tasks. Moreover, chronic diseases are commonly associated with chronic symptoms such as pain and fatigue and can have a sizeable impact on work, finances and social functioning [1].

Rates of childhood chronic disease have risen significantly over recent decades, with estimates highlighting that 1 in 4 children are affected by conditions such as asthma, gastrointestinal diseases, diabetes and cancer [6,7]. Epidemiological data demonstrate that neurodevelopment disorders have been increasingly diagnosed among children, with rates of attention deficit hyperactivity disorder (ADHD) now estimated at 5% within the United Kingdom [8]. Furthermore, approximately eleven million children under the age of fifteen are impacted by epilepsy, illustrating the variety of conditions that childhood chronic disease encompasses [9].

### 1.2. Psychological Impact and Medical Trauma

Literature has demonstrated the comorbidity of mental health difficulties and chronic disease [10]. A range of health conditions have been found to increase the risk of depression, anxiety, post-traumatic stress, and suicidal ideation while negatively impacting well-being and overall quality of life [11,12,13,14]. These outcomes are in part due to any physical difficulties associated with the condition, uncertainty about prognosis, lower levels of social integration and activity, discrimination, diminished sense of self-efficacy, poorer quality employment, reduced work hours and financial strain [15,16]. For example, Ziarko et al. [17] report that 14–62% of individuals with rheumatoid arthritis experience depression, while individuals with diabetes are more likely to experience eating disorders and anxiety disorders compared to their counterparts [18]. A recent qualitative research study, using semi-structured interviews, found that people who had undergone a permanent colostomy reported it as a significant and traumatic life change [19]. Further, living with an implantable cardioverter-defibrillator (ICD) was associated with more threatening illness perception and lower quality of life, compared with controls, in an international cross-sectional study of over 3000 adults with congenital heart disease [20]. Further, sudden cardiac arrest is associated with depression, anxiety and post-traumatic stress and the development of heart-focused health anxiety, which can undermine quality of life [21].

Traumatic stress symptoms are common among individuals following medical experiences. One meta-analysis suggests approximately 1 in 5 adults admitted to intensive care experience traumatic stress symptoms 12 months after discharge [22]. Further, a systematic review of post-traumatic stress disorder (PTSD) diagnosis in adults with cardiovascular disease found prevalence rates of up to 38%, and meta-analytic data suggest a lifetime prevalence rate of 12.6% among individuals who have had cancer [23,24].

Prevalence of psychological difficulties associated with chronic disease are common in individuals who have been chronically ill since childhood [25]. A systematic review by Secinti et al. [26] demonstrated that children who are chronically ill with conditions such as epilepsy and cystic fibrosis are at increased risk of developing emotional difficulties that persist across the lifespan into adulthood. Meentken et al. [12] reported that 12–31% of children undergoing surgery for congenital heart disease (CHD) developed post-traumatic stress disorder (PTSD). A recent comparative cross-sectional study found that 18.2% of children with CHD experienced anxiety or depression, compared with 5.2% of those without CHD [27]. Abbey et al. [24] report that post-traumatic stress disorder and post-traumatic stress symptoms are commonly experienced in adolescent childhood cancer survivors.

Aside from disease-related difficulties, a range of mental health risk factors likely contribute to these findings, including any medical and social barriers to developmental needs for secure attachment and the soothing presence of responsive caregivers, a sense of belonging, and the opportunity to play and learn [28]. Stigma, feeling different, impaired physical functioning, physical symptoms, medical tests, treatment regimens and interventions and associated losses may also contribute [12]. Further, childhood illness affects the whole family, and parents and siblings also face increased risk of mental health difficulties and impact on family dynamics [29,30]. Chronic disease in childhood is also associated with poorer academic performance and developmental, psychological and behavioural difficulties [31,32,33].

Healthcare environments and procedures can be painful and distressing for children, exacerbated by repeated exposure for those requiring chronic disease care [34]. Research suggests there is a lack of psychological safety during medical procedures such as scans, blood tests, hospital stays or surgical interventions [35], which can foster feelings of hopelessness, fear, and a lack of autonomy for individuals with chronic disease, contributing to the development of life-long psychological difficulties [16].

Despite the development of tools to assess pain in non-verbal infants and a range of interventions to reduce pain associated with medical procedures, studies suggest that pain in children is not always adequately addressed, which has been linked to lasting consequences during a critical time in brain development [36]. Further, 81% of medical professionals reported children being forcefully held, often by parents, for medical procedures ‘frequently’ or ‘very frequently’ to complete the procedure quickly despite potentially causing them to become scared of having future procedures and contributing to post-traumatic stress [37]. Feelings of powerlessness may be exacerbated by disempowering aspects of healthcare provision, such as being asked to wear a backless hospital gown [38].

Studies have shown that early stress or trauma experienced by infants and children affects neural, behavioural, and psychological development, with long-lasting effects across a wide range of domains [39]. As such, developmental medical trauma may narrow the window to feeling psychologically safe as our bodies tune to meet adversity and negatively affect the development of self-soothing strategies that enable self-regulation of emotions in later life [40]. Addressing feelings of psychological safety during medical encounters also has implications for treatment adherence and physical health outcomes. For example, children are most likely to develop a clinically impacting needle phobia because of a traumatic medical experience, influencing how much they engage with medical procedures in the future [41].

### 1.3. Polyvagal Theory as a Novel Explanatory Framework

Polyvagal Theory (PVT) offers a novel and useful framework to understand how our bodies respond to stress during healthcare encounters [42]. Grounded in neurophysiology, psychology, and evolutionary theory, PVT describes how the autonomic nervous system (ANS) subconsciously and continually assesses situations for safety or threat, termed neuroception. In situations detected via neuroception as psychologically safe, our bodies operate in the ventral vagal system of the parasympathetic nervous system, which activates physiological, affective, and cognitive processes that optimise social engagement and pro-social, compassionate behaviours. In this mode we feel calm, relaxed, and psychologically safe while our bodies are optimised for healing, learning and social connection. In situations detected as unsafe our bodies shift into self-defence mode, most commonly via activation of the sympathetic system, which leads to fight or flight behaviours that are supported by increases in heart rate, shortened breathing and increased muscle tension. During this hyperarousal, our bodies prepare to act to fight or flee the perceived danger. Finally, when we perceive a threat to life, our most primitive threat response is activated via the dorsal vagal pathway, and our bodies experience hypoarousal, often reported as feeling numb or dissociated.

Psychological safety in healthcare is essential for the well-being and mental health of chronically ill patients, especially those with early diagnoses. This is supported by research; for example, Hupcey [36] describes findings from a qualitative research study using grounded theory that explored the psychosocial needs of critically ill patients, with participants reporting an overwhelming need to feel safe. According to PVT, psychological safety is not simply the removal of threat, we constantly seek and exchange signals of safety with each other through voice tone, facial expression, body language, trust signals, reciprocity and compassion [35]. As such, compassion is fundamental to relationship-based care, helping patients feel safe and increasing engagement, and it is linked with an increase in the patient’s hope for recovery, accountability, control over their health, trust and satisfaction, leading to the provision of safer care, lowered blood pressure and pain perception, and increased survival rates and resilience of healthcare professionals [43,44,45,46,47].

### 1.4. Rationale for Study

Psychological safety during medical experiences is a crucial factor in protecting the mental health of individuals with lifelong health conditions. Despite this, there is limited research examining how individuals who have experienced chronic illness from childhood perceive psychological safety in healthcare contexts. Existing studies predominantly focus on the perspectives of healthcare providers [48], leaving the lived experiences of patients underexplored. This gap in understanding highlights the need to prioritise patient voices in research to ensure care practices are responsive to their needs.

Chronic illnesses often involve repeated medical interventions, which can be distressing and may contribute to long-term psychological difficulties, including anxiety, post-traumatic stress, and reduced health-related quality of life [49]. By identifying barriers and facilitators to psychological safety, this study seeks to inform psychologically informed, trauma-informed, patient-centred clinical practices that promote mental well-being, improve patient autonomy, and mitigate the risk of medical trauma.

This research is grounded in PVT [42], which provides a novel neurophysiological framework for understanding how individuals perceive safety and threat in healthcare settings. Using the Neuroception of Psychological Safety Scale (NPSS) [50] to guide thematic exploration, this study focuses on participants’ lived experiences to uncover factors that influence psychological safety during medical interactions.

The aims of the study are to:

- Explore which factors hinder psychological safety during medical experiences for people with a chronic illness from childhood.
- Explore which factors promote psychological safety during medical experiences for people with a chronic illness from childhood.
- Amplify patient voices by exploring their lived experiences and integrating their insights into recommendations for compassionate, trauma-informed care.
- Inform clinical practice by providing actionable insights to improve psychological safety and health-related quality of life for chronically ill individuals.

## 2. Materials and Methods

A qualitative design was employed to capture first-hand experiential accounts of the barriers and facilitators to psychological safety experienced by patients with chronic illnesses. This approach facilitated a sensitive and inductive exploration of the topic, providing participants with the opportunity to share their perspectives and lived experiences in their own words [51]. Qualitative methods are particularly well-suited to health research, as they enable a deep understanding of the patient experience, uncovering nuances that cannot be adequately captured through quantitative measures. A qualitative design grounded in an ontologically constructivist and interpretive epistemological stance was employed to explore the subjectivity of each participant’s health experiences and their personal construction of knowledge. This approach recognises that reality is socially co-constructed through language, context and social interaction, and the research seeks to understand how participants make sense of their own lived experiences [52]. Within this framework, the concept of psychological safety is understood as a subjective, context-dependent phenomenon. The interviews are therefore not treated as representations of objective ‘facts’ but as narrative accounts through which participants make sense of and give meaning to their experiences in relation to psychological safety within medical contexts.

The research questions were informed by the PVT, which guided the development of the interview protocol aimed at eliciting in-depth, narrative accounts from participants (see Table 1 and Table 2). Semi-structured interviews were selected as the primary method of data collection, as they offered the flexibility for spontaneous discussions while fostering a close rapport between the researcher and participant. This dynamic environment encouraged open and honest disclosure, enhancing the richness of the data collected [53].

### 2.1. Recruitment

Prior to recruitment, a target sample size of 6–8 participants was determined. This range was informed by guidance from Namey et al. [54], who highlight that focused, smaller samples are effective in qualitative research when participants share common, lived experiences. This size was deemed appropriate to allow for in-depth exploration of individual perspectives while maintaining a manageable volume of data for rigorous analysis. Additionally, a smaller sample ensured that the unique voices of participants could be closely examined and accurately represented, aligning with the study’s aim to amplify patient experiences.

Participants were recruited through purposive sampling, which allowed for the intentional selection of individuals with relevant experience of chronic illness from childhood. Recruitment was conducted via word of mouth and social media platforms such as X (formerly Twitter) and Instagram, where a study poster containing the researcher’s and supervisor’s contact details was shared. Health and Social Care Alliance Scotland also acted as a gatekeeper, advertising the study through their social media channels, website, and network of members. As a nationwide charity that advocates for individuals with chronic illnesses, the Alliance’s involvement helped ensure the recruitment of a suitable and diverse participant group. Interested participants were invited to contact the research team directly to express their interest and were provided with a participant information sheet and consent form to review and sign, ensuring informed consent prior to their participation.

### 2.2. Participants

A total of six participants (N = 6) were recruited for the study, ranging in age from 20 to 64 years (M = 35.5). Of the participants, four identified as female (N = 4) and two as male (N = 2); see Table 3. All participants resided in the United Kingdom. Participation was voluntary, and no financial compensation or reimbursement was provided for their time. To be eligible for the study, participants were required to meet the following criteria: they were over the age of 18, capable of providing informed consent, and had a self-reported chronic illness diagnosed before the age of 16 that necessitated medical interventions.

After 4–5 participants, there were clear themes emerging across all participants. The lead researcher then conducted a further interview (N = 6), which again confirmed the themes that were developed. At this point a decision was made to end recruitment in order to retain the depth and nuance of individual experiences of living with chronic illnesses. The sample size of six participants is justified based on the qualitative and exploratory nature of the study, which prioritises depth and richness of data over generalisability [55]. In research involving individuals living with chronic illnesses, a smaller sample allows for a more focused exploration of complex, nuanced experiences that may otherwise be overlooked. This approach is consistent with established qualitative methodologies, where smaller samples are appropriate when the aim is to generate in-depth understanding of participants lived experiences. Furthermore, previous research has demonstrated that data saturation, the point at which no new themes emerge, can be reached with six interviews, particularly when the participant group is relatively homogenous in experience [54,55,56]. Given the potential physical and emotional burden on participants managing chronic conditions, a smaller sample also ensures ethical sensitivity and allows for sustained, meaningful engagement with each individual. This enhances the overall quality and authenticity of the data collected.

The chronic conditions reported by participants included juvenile idiopathic arthritis (N = 2), diabetes (N = 1), colitis (N = 1), Tourette’s syndrome (N = 1), and congenital heart disease (N = 1).

### 2.3. Procedure

Semi-structured interviews were conducted online via Microsoft Teams version 25060.205.3499.6849, between April and June 2024. Participants were offered either an in-person interview at the university or online to increase accessibility. All participants were selected online. All interviews were recorded and transcribed using transcription software on Microsoft Teams version 25060.205.3499.6849. Recordings and transcriptions were stored on an encrypted and password-protected OneDrive account in accordance with the British Psychological Society (BPS) Code of Human Research Ethics [57]. An interview guide with four questions and follow-up prompts to facilitate discussion was prepared beforehand. The interview guide (see Table 2) was structured based on the Neuroception of Psychological Safety Scale (see Table 1), which is a psychometrically validated measure of psychological safety grounded in the Polyvagal Theory [50]. At the beginning of the interview, demographic details were noted, and consent was reaffirmed. Participants were made aware that an interview guide had been prepared, but unplanned, spontaneous discussion of different topics was encouraged. Interviews lasted between 45 min and 1 h. After the planned questions had been asked, participants were invited to add any other relevant information and then thanked for their time. Debrief forms were then emailed to participants that summarised the discussion.

### 2.4. Ethics

The study was granted ethical approval by the University Ethics Committee. In line with ethical guidance from BPS [57] and the university, participants were required to sign the consent form prior to the interview and give their verbal consent at the start of the interview. Participants were informed that the research team could not guarantee full anonymity but would take steps to protect their privacy by issuing them a pseudonym and changing identifying information such as names of people, places, and organisations. Participants were made aware that they had until one week following the interview to request the removal of their data. Only essential information relating to the study was collected, and data were stored on a secure and password-protected OneDrive account that was only accessible to the student and supervisor. Data were confidentially destroyed on submission of the assignment.

The research team were aware that participants were vulnerable due to the sensitive nature of the topics discussed. To mitigate the impact, participants were sent a debrief sheet on completion of the interview that contained contact details for support organisations and were informed prior to the interview that they could bring someone with them for moral support. The participant information sheet also helped prepare participants for the type of questions they were likely to be asked. Furthermore, the participant information sheet outlined that they were not obligated to answer questions they were not comfortable with and could revoke their consent at any time, without reason. Participants were offered regular breaks, and consent was viewed as a continual process and informally checked on by being aware of facial expressions and body language that signalled stress. To minimise researcher bias and fatigue, the interview guide was checked by the supervisor beforehand, and the analysis was checked and compared with a colleague.

### 2.5. Analysis

Following the interviews, transcripts were edited verbatim and analysed using an inductive, reflexive thematic analysis [52]. This approach was chosen for its systematic and transparent process, enabling the identification of themes and meanings within the data [56]. The analysis adhered to Braun and Clarke’s [58] six-step process, beginning with familiarisation through repeated readings of the transcripts to identify potentially relevant content. A critical re-reading was then conducted to code the data, capturing its analytical essence. These codes were refined, grouped, and developed into candidate themes. Themes were reviewed for coherence, further refined, and named to accurately reflect the dataset. Representative quotes were selected to ground each theme in the participants’ narratives, allowing for a rich and nuanced understanding of their lived experiences.

In accordance with Braun and Clarke’s [55] reporting guidelines for qualitative research, the analysis was guided by a value-based approach that supports the evaluation and reporting of qualitative research on its own terms. This framework prioritises qualitative research values, such as methodological congruence, reflexivity, and openness, to ensure alignment between the chosen methodology and the epistemological and theoretical underpinnings of the research. This study upheld these principles by ensuring that the use of thematic analysis was congruent with the ontological and epistemological stance of exploring participants’ subjective experiences. Reflexive openness was maintained throughout the research process, with the researcher critically reflecting on their positionality and the potential influence of their assumptions on the analysis. The study also emphasised rich and contextual reporting to honour the complexity and depth of participants’ experiences. Themes were presented with representative participant quotes to foreground their voices and ensure that the findings authentically reflected their narratives. Care was taken to avoid methodological incongruence by ensuring that the analysis and reporting processes were not constrained by criteria misaligned with qualitative values.

To enhance transparency and trustworthiness, the research adhered to principles aligned to the Consolidated Criteria for Reporting Qualitative Research (COREQ). An audit trail was maintained throughout the analysis, providing a detailed record of each stage, including the generation and refinement of codes, the development of themes, and decision-making processes. Regular consultations with the co-researchers ensured that the analysis was rigorous and credible, strengthening the reliability of the findings. By integrating Braun and Clarke’s [55] value-based framework with established qualitative reporting standards, the study ensured a robust, reflexive, and transparent approach to analysis and reporting.

### 2.6. Reflexivity

Both the principal investigator (HR) and co-researcher (LM) share personal experiences of living with chronic health conditions from childhood. This shared identity afforded a unique insider perspective, facilitating a deeper understanding, relevance and empathy of the issues faced by participants. However, it also required careful reflection to balance the potential for empathic understanding with the need to remain critical and objective. As both “insiders” and “outsiders” in the research process, the researchers were mindful of how their personal experiences as individuals with chronic conditions might influence both their interpretations and their relationships with participants. For example, HR’s position as someone with a lived experience of chronic illness created a common ground for participants, which could foster trust and openness. However, it also carried the risk of imposing her own understanding or bias into the participants’ narratives. To mitigate undue influence and ensure analytical rigour, we engaged in reflexive journaling, regular team debriefs, and critical discussion during the theme development process. These strategies were employed to foster transparency and credibility throughout the study.

Specifically, HR maintained a reflexive journal throughout the data collection and analysis process to document reflections, enhancing transparency and minimising bias in the interpretation of findings. The journal was used not only to capture personal thoughts and reactions to the interviews but also to track how the lead researcher’s (HR) experiences and beliefs could shape her interpretations. By maintaining a reflective journal, HR was able to capture not only her emotional responses but also the nuances of her interactions with participants, providing insight into how her own experiences might influence data collection and analysis. This ongoing self-awareness was essential in managing the complex interplay between insider and outsider perspectives, ensuring that the research was ethically sound, reflective, and transparent. This practice helped HR to consciously set aside her assumptions, ensuring that the voices of the participants remained central to the findings.

HR also considered whether to disclose her own chronic health condition to participants. After discussions with the research team, it was agreed that if the opportunity naturally arose during the interview, she would disclose her condition in order to build rapport and equalise the power dynamic between the interviewer and participant. This decision was made with the intention of reducing hierarchical tensions and fostering a more open and collaborative atmosphere during the interviews. HR was aware that some participants might withhold information for fear of upsetting her, so she encouraged them to answer as fully as they felt comfortable, regardless of her disclosure. The disclosure occurred naturally with three of the six participants, where it helped to build a sense of alliance and solidarity, contributing to a deeper, more authentic dialogue.

## 3. Results

The analysis of interviews identified four main themes capturing the key barriers and facilitators to psychological safety during medical procedures for individuals diagnosed with chronic illness in childhood. These themes that developed were (1) knowledge empowerment through information and facilitated inquiry, (2) holistic acknowledgment of psychological and social impacts, (3) the role of parental involvement in healthcare interactions, and (4) the need for an individualised, patient-centred approach (see Table 4).

### 3.1. Theme 1: Knowledge Empowerment Through Facilitated Inquiry

Participants expressed that a sense of empowerment through knowledge significantly contributed to their feelings of psychological safety. Having access to clear information about their condition and procedures fostered a sense of control and confidence, while the lack of information often resulted in fear and helplessness. Participants who received comprehensive explanations about their condition and procedures felt better prepared and more psychologically safe. Conversely, a lack of information during childhood led to confusion and stress.

“I just don’t think that was explained to me that… you will feel them especially for the first ten years of being a diabetic … even when I was having these problems of sleeping at night, I don’t remember anyone’s explicitly telling me ‘no, you will feel them.’”

(Tim)

Tim’s lack of understanding about his diabetes symptoms fostered fears that later manifested as obsessive-compulsive behaviours. Similarly, Archie felt isolated due to insufficient information about his condition:

“I’d never heard of the illness when I got it … it would have been great to have been informed that you know there were other people that suffer with it… I think I probably didn’t need to feel so ashamed.”

(Archie)

Without knowing that ulcerative colitis affected others, Archie felt isolated and ashamed, which negatively impacted his social interactions and self-image.

Participants emphasised that the opportunity to ask questions about their care was a major factor in feeling psychologically safe. However, many recalled limited opportunities for open dialogue with healthcare providers.

“Doctors didn’t actually tell you what was going on. They would ask you questions, and you were never encouraged to ask questions. They would tell you … and that was it.”

(Paula)

Paula felt that the lack of encouragement to ask questions was disempowering, reinforcing a sense of passivity in her care. Julie shared how the adult–child power dynamic deterred her from asking questions, despite her mother’s encouragement:

“My mammy always would say ‘have you got any questions? If you’ve got any you just ask.’ But you wouldn’t ‘cause it was an adult… Whereas now I will ask lots of questions and ask them to draw diagrams … so I know how to cope; that kind of knowledge is power.”

(Julie)

As an adult, Julie now views knowledge as an essential coping tool, underscoring the value of encouraging open dialogue with young patients.

### 3.2. Theme 2: Holistic Acknowledgment of Psychological and Social Impacts

Participants felt that healthcare providers often focused solely on their physical health, neglecting the broader psychological and social impacts of chronic illness. This narrow focus often left participants feeling unsupported in managing the full scope of their experiences.

Participants highlighted the need for mental health support alongside physical treatment. They expressed disappointment in the lack of psychological support during diagnoses and treatment.

“I think it was crazy that there was never a psychologist in the room … you’ve just told a kid that they have a chronic illness. Their life is gonna change or had already changed.”

(Tina)

Tina was struck by the absence of mental health support during her diagnosis, suggesting that early integration of psychological care could improve long-term outcomes. Julie similarly described how repeated hospitalisations impacted her mental state, emphasising the need for emotional support:

“I was in hospital for something, and I ran and the nurse had to chase me downstairs and got a hold of me … the hospital had playrooms and stuff, but I didn’t want them. I just wanted home.”

(Julie)

Julie’s reaction reflects a stress response, indicating her heightened need for psychological support during prolonged hospital stays.

Participants noted that their chronic illness often limited social interaction, yet this aspect was rarely acknowledged or addressed by healthcare providers. This lack of support for social challenges left some participants feeling alienated.

“I would … completely avoid all social interaction with people that I didn’t feel totally comfortable ticking in front of. It was like I’ll just put myself in my room and just don’t deal with it.”

(Grace)

Due to the stigma associated with her condition, Grace often isolated herself socially, a behaviour she attributed to the lack of psychological and social support during her youth. Julie also shared how her condition affected her ability to socialise:

“I couldn’t go out to play, I tired very easily. I didn’t really have a lot of social interaction like with other children. I think everybody kinda had labelled you the kid with … the bad heart… You stop being a person to an extent.”

(Julie)

This quote illustrates how labelling and limited socialisation can have lasting effects on identity and psychological well-being, underscoring the need for healthcare providers to acknowledge these broader impacts.

### 3.3. Theme 3: The Role of Parental Involvement in Healthcare Interactions

Participants described parental involvement as both a support and, at times, a limitation. While parents often provided a buffer against the challenges of navigating healthcare, their involvement sometimes led to difficulties when participants transitioned to managing their care independently. For many participants, having parents communicate with doctors provided a sense of safety and reduced the stress associated with complex medical interactions.

“As a child I wouldn’t really like to speak anyway, like my parents probably did most of speaking on my behalf. My mum anyway.”

(Tina)

Tina’s experience reflects the protective role her mother played, reinforcing her psychological safety by advocating on her behalf. While parental involvement was beneficial during childhood, some participants struggled with managing their condition independently as adults due to abrupt transitions.

“I turned 18 and my mum was like ‘you now need to start dealing with your own appointments’ … I found it so overwhelming because I went from ‘I just need to show up’ to now ‘I actually take charge of like my medication … it wasn’t like you were kind of like phased into it. It was just like ‘right you’re 18 you gotta deal with it.’”

(Grace)

Grace felt overwhelmed by the sudden responsibility for her health, highlighting the need for gradual transitions in responsibility. Tim, however, felt that his parents’ strong involvement hindered his autonomy:

“My parents were quite pushy for me to go on the pump. Sort of a lot of them going ‘oh you should do this’ … and me going ‘look I just want to do my thing’”

(Tim)

Tim’s experience demonstrates the importance of respecting young patients’ preferences, especially as they approach adulthood.

### 3.4. Theme 4: Need for an Individualised, Patient-Centred Approach

Participants emphasised the value of a personalised approach to care, with healthcare interactions tailored to individual needs and preferences. Psychological safety was enhanced when participants felt their unique perspectives were respected.

Participants noted that the ability to have control over aspects of their care, even in small ways, was key to feeling psychologically safe. For instance, Tim described a technique used by his diabetes nurse to help him manage injections:

“The way we did it was they held it in place but I pushed the buttons for it then to go in so I was in control … I’ve always had to be in control of the needle.”

(Tim)

Tim’s sense of control over his injection process helped him manage his anxiety, illustrating how small adjustments can enhance psychological safety. Julie and Paula had contrasting preferences for conversation during procedures, reflecting the individuality of psychological safety needs. Julie preferred minimal interaction:

“I had one gentleman try to talk to me … I said ‘like I can’t talk … and it’s not you, it’s just the process’ … I want this done and out. I’m not here…for idle chit chat.”

(Julie)

In contrast, Paula found that casual conversation with her doctor helped her feel calm:

“I was so anxious because I didn’t know what he was going to tell me so he spent the first ten minutes just chatting about my family, chatting about things … and he just put me ease. I could feel myself kind of, you know, and breathe (mimics motion of breathing) so that I could actually then listen to what he was actually telling me.”

(Paula)

The results highlight the importance of clear communication, psychological support, and a personalised, patient-centred approach in fostering psychological safety. Participants felt empowered when provided with comprehensive information and the opportunity to ask questions, while a lack of support for the psychological and social impacts of chronic illness often led to feelings of isolation and stress. Parental involvement was seen as both helpful and a barrier to independence, with some struggling to manage care alone as they transitioned into adulthood. Overall, participants emphasised the need for healthcare that acknowledges both physical and emotional needs, offering tailored care that respects individual preferences.

## 4. Discussion

This study sought to understand the barriers and facilitators to psychological safety experienced by individuals with chronic illnesses during medical procedures, amplify their voices, and provide insights to inform compassionate, psychologically informed healthcare [59]. By exploring the lived experiences of participants, the study highlights actionable recommendations for improving psychological safety and health-related quality of life in clinical practice. Thematic analysis revealed four key themes—knowledge empowerment, holistic acknowledgment of psychological and social impacts, the role of parental involvement, and the need for individualised, patient-centred care—each addressing critical aspects of the study’s aims.

Participants identified access to clear, comprehensive information as a key facilitator of psychological safety. Being well-informed about medical procedures empowered them to feel more in control and less fearful, reflecting the importance of health literacy in promoting confidence and reducing distress [60]. Participants noted that developmentally appropriate communication during childhood could have mitigated psychological difficulties, such as feelings of isolation and anxiety. Encouraging open dialogue and providing opportunities for patients to ask questions further enhanced their sense of safety, aligning with the increasing emphasis on patient-led healthcare practices such as shared decision-making and rights-based care [61,62]. These findings underscore the necessity of integrating compassionate communication [47] strategies that prioritise knowledge empowerment into clinical practice to enhance patient experiences and foster feelings of psychological safety.

Despite these facilitators, participants identified significant barriers to psychological safety, particularly the lack of acknowledgment by healthcare providers of the broader psychological and social impacts of chronic illness. Participants described feelings of loneliness, stigma, and disconnection, particularly during childhood when emotional support was often lacking. These experiences reflect a failure to adopt a holistic, biopsychosocial approach to care that recognises the interplay between physical, psychological, and social health [63]. The findings support the integration of psychologically informed and trauma-informed healthcare practices that address these wider impacts, helping to foster psychological safety and mitigate long-term mental health difficulties.

Polyvagal Theory [42] provides a novel and useful framework for understanding how feelings of disconnection and isolation may reflect the activation of defensive physiological states, highlighting the importance of creating environments that promote safety and engagement. It is important to note that while PVT has received broad consensus in scientific literature, like any theory, it has also attracted criticism [64]. Porges has provided extensive rebuttal to critiques [65]. For example, in response to a contention that PVT incorrectly subscribes to a false dichotomy between asocial reptiles and social mammals [66] Porges notes that these criticisms are irrelevant to PVT, which is mammal-centric. Specifically, sociality through a polyvagal lens focuses on the transformative qualities of social behaviour expressed in mammals and not observed in reptiles (e.g., nursing during mother–infant interactions, vocal intonations, facial expressions, and other co-regulatory behaviours) that impact on calming the ANS to optimise homeostatic functions.

In the current study, the role of parental involvement emerged as both a facilitator and a potential barrier to psychological safety. Parents often acted as advocates and buffers, providing critical support in managing appointments and communicating with healthcare professionals during childhood. However, participants expressed distress when transitioning to adult services, where parental involvement was abruptly reduced. This highlights the importance of facilitating gradual transitions to self-management to support autonomy and minimise distress [67,68]. Conversely, some participants welcomed the opportunity to take greater control of their healthcare decisions as they matured, reinforcing the need for healthcare providers to balance parental involvement with respect for the patient’s developing independence [69,70]. These findings emphasise the value of family-inclusive yet patient-centred care models that adapt to the evolving needs of chronically ill individuals over time.

An individualised approach to care was identified as critical to fostering psychological safety. Participants highlighted the importance of feeling respected and having control over their medical procedures, with preferences varying widely between individuals. For example, some participants preferred active involvement in their care, while others found reassurance in minimising interaction during procedures. This variability underscores the inadequacy of standardised approaches to care and supports the adoption of tailored, patient-centred practices that align with individual preferences [71]. By recognising and accommodating the unique needs of each patient, healthcare providers can create environments that promote psychological safety and enhance trust and engagement [16].

### 4.1. Strengths and Limitations

This study has several strengths that contribute to its significance. By using a qualitative design grounded in thematic analysis, it provides rich, in-depth insights into the lived experiences of individuals with chronic illnesses diagnosed in childhood. The use of Braun and Clarke’s [55] value-based reporting guidelines ensured that the analysis was methodologically congruent and reflexively transparent, capturing nuanced perspectives while maintaining research rigour. Additionally, the study highlights the voices of a population whose experiences of psychological safety in healthcare contexts are often underexplored, contributing valuable knowledge to an area of growing clinical importance. The incorporation of participant narratives and the alignment of findings with existing research enhance the study’s credibility and relevance to improving patient-centred and trauma-informed care practices.

However, the study also has limitations. The sample was entirely UK-based, which restricts the transferability of findings to other healthcare systems and cultural contexts. Furthermore, all participants identified as white, and the majority were female, which limits the applicability of the findings to more diverse populations, particularly in light of documented disparities in healthcare access and outcomes across ethnic and gender groups [72]. The small sample size, while appropriate for qualitative research, reduced the breadth of experiences represented and may not fully reflect the diversity of chronic illness trajectories. Additionally, the reliance on self-reported data introduces the potential for recall bias, as participants’ reflections may be influenced by memory limitations or personal framing of past events. The absence of respondent validation further limits the ability to confirm the accuracy of the findings with participants, which could have strengthened the trustworthiness of the data. Further transferability is limited by a lack of information about potentially confounding variables such as socio-economic status and exposure to other adverse childhood experiences or traumatic experiences that may impact feelings of psychological safety. Despite these limitations, the study provides an important foundation for future research and actionable insights for clinical practice, particularly in promoting psychological safety through patient-centred, trauma-informed approaches.

### 4.2. Directions for Future Research

Future research should address these limitations by incorporating more diverse samples in terms of ethnicity, gender, and cultural context to capture a broader range of experiences. Further, triangulation of the findings could be sought, for example, by linking self-reports to other sources such as medical records or completion of the Neuroception Psychological Safety Scale (NPSS) during healthcare encounters. Longitudinal studies could explore how psychological safety evolves over time, particularly as patients transition from paediatric to adult care. Further investigation into the role of parental involvement and strategies for facilitating smooth transitions to self-management could provide valuable insights for improving care delivery. Finally, research should evaluate the effectiveness of trauma-informed and patient-centred interventions in promoting psychological safety, with a focus on integrating these approaches into routine clinical practice [73].

## 5. Conclusions

Psychological safety is a multifaceted and individual experience, shaped by a variety of factors. This qualitative study, through semi-structured interviews and thematic analysis, explored the barriers and facilitators to psychological safety during medical procedures for individuals who have lived with chronic illness since childhood. The findings demonstrate that psychological safety is influenced by the patient’s knowledge, the role of parental involvement, the level of individualisation in care, and whether healthcare professionals acknowledge the wider psychological and social impacts of chronic disease.

The study highlights the essential role that psychological safety plays in promoting mental health and well-being. For individuals with chronic conditions, feeling safe in healthcare settings can prevent or reduce the development of mental health difficulties, such as anxiety, depression, and PTSD, which are often linked to traumatic medical experiences. In clinical practice, the findings emphasise the need for psychologically and trauma-informed care that prioritises clear communication, patient empowerment, and the involvement of family members where appropriate. Additionally, this study underlines the importance of individualised, patient-centred approaches to care, where healthcare providers recognise the unique experiences of each patient. Empowering patients with information, encouraging open dialogue, and respecting individual preferences during medical procedures are key to fostering psychological safety. These practices can help mitigate fear, improve patient satisfaction, and ultimately contribute to better health outcomes. Incorporating these findings into clinical practice can guide healthcare professionals in creating safer, more supportive environments for patients with chronic illnesses, improving their overall experience and long-term mental health. The results underscore the importance of considering both the physical and emotional needs of patients, ensuring that healthcare practices are responsive to the complex realities of living with chronic illness.

## Figures and Tables

**Table 1:** Items on the Neuroception of Psychological Safety Scale (NPSS) [50].

1	I felt valued	1	2	3	4	5
2	I felt comfortable expressing myself	1	2	3	4	5
3	I felt accepted by others	1	2	3	4	5
4	I felt understood	1	2	3	4	5
5	I felt like others got me	1	2	3	4	5
6	I felt respected	1	2	3	4	5
7	There was someone who made me feel safe	1	2	3	4	5
8	There was someone that I could trust	1	2	3	4	5
9	I felt comforted by others	1	2	3	4	5
10	I felt heard by others	1	2	3	4	5
11	I felt like people would try their best to help me	1	2	3	4	5
12	I felt cared for	1	2	3	4	5
13	I felt wanted	1	2	3	4	5
14	I didn’t feel judged by others	1	2	3	4	5
15	I felt able to empathise with other people	1	2	3	4	5
16	I felt able to comfort another person if needed	1	2	3	4	5
17	I felt compassion for others	1	2	3	4	5
18	I wanted to help others relax	1	2	3	4	5
19	I felt like I could comfort a loved one	1	2	3	4	5
20	I felt so connected to others I wanted to help them	1	2	3	4	5
21	I felt caring	1	2	3	4	5
22	My heart rate felt steady	1	2	3	4	5
23	Breathing felt effortless	1	2	3	4	5
24	My voice felt normal	1	2	3	4	5
25	My body felt relaxed	1	2	3	4	5
26	My stomach felt settled	1	2	3	4	5
27	My breathing was steady	1	2	3	4	5
28	I felt able to stay still	1	2	3	4	5
29	My face felt relaxed	1	2	3	4	5

**Table 2:** Interview schedule derived from the NPSS.

Questions	Further Prompts
(1) To start us of could you tell me a bit about your chronic disease, perhaps when you were diagnosed, what your symptoms were like?	What was the diagnosis process like for you?
(2) What kind of medical procedures do you need to undergo for your condition?	During these procedures, how psychologically safe do you feel within your body. Can you describe some of the sensations? How was your breathing? How did your heart rate feel? Did you feel relaxed? Did your stomach feel settled? Were you able to stay still? Can you tell me what impacted on this? What was your relationship with your body like during this time? Could you tell me a bit about how you felt about your capacity for social interactions during this time? Did you feel comfortable expressing yourself? Did you feel like you could empathise with others? Did you feel able to comfort others? Did you feel connected to others? Did you feel like you could care for others during that time? Can you tell me what impact on this? What was your interactions and relationship to others in your life like during this time? Did you feel cared for? Did you feel understood? Did you feel wanted? Was there someone you felt safe with? Can you tell me what impacted on this?
(3) Can you recall some of your earliest experiences of treatments of procedures you experienced?	How did that make you feel? Who accompanied you? During these early procedures, how psychologically safe do you feel within your body. Can you describe some of the sensations? How was your breathing? How did your heart rate feel? Did you feel relaxed? Did your stomach feel settled? Were you able to stay still? Can you tell me what impacted on this? What was your relationship with your body like during this time? Could you tell me a bit about how you felt about your capacity for social interactions during this time? Did you feel comfortable expressing yourself? Did you feel like you could empathise with others? Did you feel able to comfort others? Did you feel connected to others? Did you feel like you could care for others during that time? Can you tell me what impact on this? What was your interactions and relationship to others in your life like during this time? Did you feel cared for? Did you feel understood? Did you feel wanted? Was there someone you felt safe with? Can you tell me what impacted this? How does this compare to a recent medical procedure?
(4) Generally thinking about undergoing medical procedures, what else helps you to feel safer or what would you like to change about the experience to help this?	Are there any particular coping strategies you use?

**Table 3:** Demographic information about participants.

Pseudonym	Condition	Age	Ethnicity	Gender
Paula	Juvenile Idiopathic Arthritis	64	White Scottish	F
Tina	Juvenile Idiopathic Arthritis	24	White Welsh	F
Tim	Diabetes	20	White Scottish	M
Archie	Colitis	30	White English	M
Grace	Tourette’s Syndrome	22	White Scottish	F
Julie	Congenital Heart Disease	53	White Scottish	F

**Table 4:** Thematic table.

Theme	Description	Key Insights
*1. Knowledge Empowerment through Facilitated Inquiry*	Access to clear, comprehensive information allows patients to feel more in control and confident in managing their conditions, fostering a sense of psychological safety. When information is limited or unclear, patients often experience feelings of fear and helplessness. Encouraging patients to ask questions and actively participate in discussions about their care enhances their understanding and engagement, helping them to better manage their health and reducing anxiety.	Patients who felt informed and able to ask questions had a stronger sense of empowerment and psychological safety. Those with limited information often experienced confusion and stress, impacting their ability to engage confidently in their care.
*2. Holistic Acknowledgment of Psychological and Social Impacts*	Beyond physical health, patients benefit from healthcare that recognises and addresses the psychological and social impacts of chronic illness. Patients reported feeling unsupported when these aspects were overlooked, leading to feelings of isolation, stress, and challenges in social situations. Incorporating mental health support from the time of diagnosis and throughout treatment helps patients manage the emotional impact of their conditions and fosters a more comprehensive approach to care.	Patients noted that while physical care was provided, limited psychological or social support led to feelings of loneliness, stigma, and difficulty adjusting socially. Recognising and addressing these aspects holistically could reduce emotional distress.
*3. Role of Parental Involvement in Healthcare Interactions*	Parental involvement can serve as an important source of support, particularly for young patients navigating complex healthcare interactions. However, sudden shifts in responsibility as they reach adulthood can be overwhelming if a gradual transition is not facilitated. Empowering young patients to gradually take on responsibility, with appropriate support from parents and providers, helps foster independence and confidence in managing their health as adults.	Parental support was often vital in childhood, yet patients felt challenged by abrupt transitions to independent care. A gradual increase in patient responsibility during adolescence could better support autonomy and readiness for adult care.
*4. Need for an Individualised, Patient-Centred Approach*	Patients benefit when healthcare interactions are tailored to their unique needs and preferences, fostering a sense of psychological safety. Individual preferences for control, communication, and comfort during care can vary significantly; understanding and accommodating these needs allows for a more positive experience. Personalised approaches, even in small adjustments, enable patients to feel respected and understood, strengthening their overall well-being.	Acknowledging each patient’s specific preferences and needs contributed to their sense of comfort and psychological safety. Individualised approaches allowed patients to feel more respected and at ease, underscoring the value of personalised, patient-centred care.

## Data Availability

The data generated and analysed during this study are not publicly available due to privacy and ethical restrictions. However, de-identified data may be available from the corresponding author upon reasonable request and with appropriate ethical approvals.
